# Uterine Leiomyoma Journey With Acessa ProVu System Using General Anesthesia Under Laparoscopic Ultrasound Guidance: A Case Report

**DOI:** 10.7759/cureus.54006

**Published:** 2024-02-11

**Authors:** Merlin Perez Navarro, Juliana Cazzaniga, Benny Esquenazi

**Affiliations:** 1 Medicine, Florida International University, Herbert Wertheim College of Medicine, Miami, USA; 2 Obstetrics and Gynecology, Memorial Healthcare, Pembroke Pines, USA

**Keywords:** general anesthesia, acessa provu, anesthesia, vascular leiomyoma, ultrasound-guided, fibroid uterus

## Abstract

This case report details the evaluation and management of a 40-year-old woman presenting with heavy menstrual bleeding and severe dysmenorrhea. Despite three months of combined oral contraceptives, symptoms persisted. The patient denied other systemic symptoms, with no weight loss, fatigue, or urinary/fecal symptoms. No visual, cardiovascular, pulmonary, abdominal, neurological, or mental health issues were reported. Pelvic imaging revealed a 7 cm × 4.3 cm FIGO 4 fibroid. The chosen treatment was laparoscopic radiofrequency ablation (Acessa) due to its efficacy, safety, and faster recovery. The case highlights the importance of a comprehensive approach to diagnosing and treating abnormal uterine bleeding.

## Introduction

A 40-year-old G 2 P 1-0-1-1 woman with a significant history of hypertension and abnormal uterine bleeding (AUB) presented with a one-year history of heavy menstrual bleeding lasting eight days, accompanied by severe dysmenorrhea for the first four days. Despite being on combined oral contraceptives (COCs) for three months, symptoms persisted. Pelvic ultrasound revealed a 7 cm × 4.3 cm intramural fibroid in the anterior left aspect of the uterus. A saline-infused sonogram showed no intracavitary component.

Physical examination revealed a palpable uterine mass. The patient denied other symptoms, with no weight loss, fatigue, or urinary/fecal symptoms. No visual, cardiovascular, pulmonary, abdominal, neurological, or mental health issues were reported. The patient had a history of hypertension for five years, and no significant surgical history. Medications included Nextstellis (Drospirenone 3mg, estetrol 14.2mg) and Lisinopril (5mg). Menarche at 13, regular periods (30-32 days) with heavy flow. The patient’s current contraceptive was Nextstellis. The patient had one pregnancy, one living child from a normal vaginal delivery 20 years ago. The last pap smear was two years ago and was normal. The patient has had a stable relationship for 13 years, with no history of tobacco, alcohol, or drug use. Family history included hypertension in the mother and colon cancer in the maternal uncle. The patient’s vitals were stable, general appearance, skin, head/neck, cardiovascular, pulmonary, abdominal, and neurological exams were unremarkable. A gynecological exam revealed a palpable uterine mass.

The patient was diagnosed with uterine leiomyoma based on history, physical exam, and imaging findings. The patient was treated with a laparoscopic radiofrequency ablation (Acessa) due to its effectiveness, safety, and faster recovery. The 7 cm fibroid size falls within the safe range for laparoscopic RFA. The Acessa ProVu system was developed by Hologic- GYN Surgical Solutions. Hologic is headquartered in Marlborough, Massachusetts, and it is a global medical technology company that offers cutting-edge solutions to facilitate accurate detection and treatment of gynecological conditions. Acessa utilizes radiofrequency currents for leiomyoma reduction [1]. The machine contains laparoscopic and intra-abdominal ultrasounds that help locate fibroids, visualize anatomy, and confirm the safety of the procedure. The Acessa handpiece tip contains seven needle-like arrays deployed once in contact with the target fibroid [1]. The system then automatically calculates the dimensions of the treatment zone and the duration of the ablation [1-3]. Radiofrequency energy is emitted through the arrays, which causes thermal destruction of the fibroid through coagulative necrosis. Studies show decreased menstrual blood loss, size reduction, and improved quality of life with the use of Acessa Adverse events are minimal, and it is a viable option for future pregnancies. Open abdominal myomectomy was considered less favorable due to increased risks, prolonged recovery, and comparable effectiveness. Laparoscopic RFA (Acessa) is the preferred treatment for this patient with uterine leiomyoma, offering a balance of efficacy and safety, aligning with the patient's preferences and lifestyle [2,3].

AUB is a common gynecological concern, that impacts women's quality of life. This case presents a 40-year-old woman with a significant history of hypertension seeking further evaluation for heavy menstrual bleeding and dysmenorrhea. Despite initial management with COCs, persistent symptoms prompted a thorough investigation, leading to the diagnosis of a uterine leiomyoma. This report explores the patient's history, examination findings, and the rationale behind the chosen treatment.

## Case presentation

**Figure 1:**
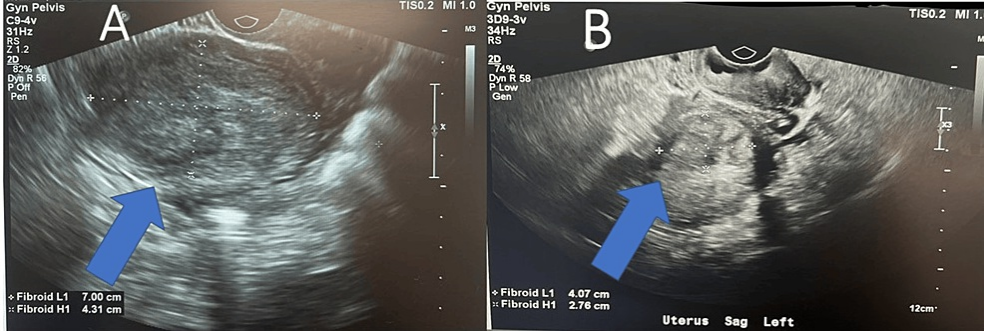
Ultrasound before and after procedure (A) Transvaginal image of pelvic organs with sonohystogram demonstrates fluid-filled endometrial cavity with catheter and saline water, negative for polyps or submucosal fibroids. Left lateral anterior intramural fibroid 7.00 cm x 4.31 cm depicted with a blue arrow and white cross on top of the fibroid to demonstrate the height and width. (B) After the procedure, the left lateral anterior intramural fibroid shrieked to 4.07 cm x 2.76 cm depicted with a blue arrow and white cross on top of the fibroid to demonstrate the height and width. The ultrasound machine used in the office to visualize pelvic organs was the Philips EPIQ 5 Ultrasound system. A transvaginal transducer was utilized to perform the saline-infused sonogram. The EPIQ 5 system was launched in 2013 and it features improved imaging technology. This includes the MaxVue high-definition display which allows extraordinary visualization of anatomy with 1,179,648 additional image pixels compared to a standard 4:3 display format mode.

The patient, a G1P1001 woman, reported a one-year history of prolonged and heavy menstrual bleeding accompanied by severe dysmenorrhea. Despite the initiation of combined oral contraceptives, symptoms persisted. Pelvic ultrasound revealed a 7 cm × 4.3 cm intramural fibroid shown in Figures 1A, 1B, prompting consideration of various differentials, including uterine fibroid, adenomyosis, endometrial cancer, and pregnancy.

The patient’s AUB persisted despite a three-month trial of COCs. Pelvic ultrasound revealed a 7 cm × 4.3 cm intramural fibroid, prompting consideration of various differentials, including uterine fibroid, adenomyosis, endometrial cancer, and pregnancy. Physical examination confirmed a palpable uterine mass. The final diagnosis was uterine leiomyoma. The treatment of choice was laparoscopic radiofrequency ablation (Acessa), chosen for its effectiveness and patient-specific advantages.

The patient was taken to the operating room where she was given general anesthesia. Induction of general anesthesia was accomplished using the inhaled anesthetic sevoflurane with MAC of 4.3, propofol 1.5mg/kg IV, ketamine 2mg/kg IV, and fentanyl 50mcg IV. Neuromuscular blockade was achieved by the use of rocuronium bromide 0.6mg/kg IV. Tracheal intubation was successful using a Mac 3m laryngoscope; the patient was a Cormack-Lehane grade 1.

She was placed in lithotomy, prepped, and draped in the usual fashion. Time out was done. Antibiotics were given. A Foley catheter and a HUMI uterine manipulator were inserted, which was insufflated with 5 cc of saline. Attention was then turned to the abdomen where a 5-mm umbilical incision was made. A bladeless trocar was passed intraperitoneally. Accessory 11 mm trocar was placed in the midline approximately 5 cm under the umbilicus.

A laparoscopic diagnostic intra-abdominal ultrasound was performed to evaluate the uterus with the previously mentioned fibroids. From side to side, the uterus was mapped, and all pertinent findings were noted. The leiomyoma was characterized by type and measured into separate perpendicular planes, both transverse and sagittal, and anterior posterior. Once the uterus was mapped, a stab wound incision was made into the laparoscopic port to allow for the handpiece to be inserted through the avascular area along the anterior abdominal wall with a minimum of 3 cm from the ultrasound port.

The leiomyoma was targeted, and measurements were taken. The probe was then inserted into the leiomyoma using ultrasound guidance, stabilizing the uterus with the uterine manipulator. Care was taken that the handpiece passed through an avascular area and did not overshoot the extent of the myoma. Confirming adequate placement, the handpiece tip was kept in ultrasound view at all times during the myometrial leiomyoma placement. The electrodes were then extended to adequately cover the extent of the myoma. This data was then used the ablation treatment algorithm for T-size uterus was estimated and the radiofrequency ablation of these fibroids was performed. Continuous temperature monitoring of all seven needles was reviewed at all times and averaged 90°C. The return pad temperature was noted to be below 40°C at all times and, following the treatment, the electrodes were retracted. The system was switched to 80-watt coagulation as the probe was slowly withdrawn to cauterize the track. A similar fashion was used to do the rest of the myomatous. After the ablation of all identifiable and accessible myomas, the handpiece was removed from the abdomen, and irrigation of the pelvis was performed. Hemostasis was confirmed laparoscopically and was excellent. Hemodynamic stability was maintained throughout the procedure and no adjunct agents were needed.

The instruments were removed from the abdomen. The abdomen was deflated after the instruments were removed. Deep breaths were given by the anesthesiologist. Surgical glue was used to cover the incision site. The Foley and uterine manipulators were removed from the vagina. The choice of neuromuscular blockade reversal agent was Sugammadex 2mg/kg IV. The patient was then successfully extubated, awakened, and transferred to PACU in stable condition.

## Discussion

The differential diagnoses included uterine fibroid, adenomyosis, endometrial cancer, and pregnancy [4]. Through a systematic approach involving history, physical examination, and imaging studies, uterine leiomyoma was identified as the cause of the patient's symptoms [4]. The decision-making process for treatment considered alternative options, such as open abdominal myomectomy, but laparoscopic radiofrequency ablation was favored due to its overall efficacy, safety profile, and quicker recovery [4,5]. The discussion emphasizes the significance of tailored treatment plans based on individual patient factors and preferences [4,5].

This patient is a 40-year-old G1P1001 LMP 7/1/23 with a significant history of hypertension and AUB that presents to the clinic for further evaluation of heavy menstrual bleeding with severe dysmenorrhea. For the past year, the patient reports regular periods that last eight days with heavy bleeding and associated severe dysmenorrhea for the first four days. She was started on COC pills three months ago, but her symptoms have not significantly improved. To evaluate her AUB, a pelvic ultrasound was performed, which revealed an intramural fibroid measuring approximately 7 cm × 4.3 cm in the anterior left aspect of the uterus. A saline-infused sonogram was performed afterward, and it showed no intracavitary component. In today’s physical exam, there was a palpable mass in the uterus and no other significant findings. Based on history, physical exam, and studies done, my final diagnosis for this patient’s AUB is uterine leiomyoma.

Laparoscopic radiofrequency ablation of leiomyoma (Acessa) is the treatment of choice for this patient based on overall effectiveness, indication based on the location and size of her fibroid, and decreased rates of adverse events and faster recovery time [5-7]. Acessa was FDA-approved in 2012 and it is a relatively new procedure that providers are starting to become more involved and experienced with [5]. The procedure uses radiofrequency currents to decrease the size of leiomyomas via laparoscopy [5]. A narrative review published in Green Journal showed that the majority of women in studies experienced a reduction in menstrual blood loss and leiomyoma size by three months after treatment, resulting in improved quality of life. Studies demonstrated the total mean myoma volume was reduced by 39.8% at three months, with a final reduction of 45.1% at one year. Since the size of this patient’s fibroid is 7cm, a 45% reduction in size would cause a significant change because she would be left with a small fibroid that could potentially be asymptomatic and is likely to further decrease or disappear after menopause. The size of her fibroid is also within the range (1-10cm) for which safe use of laparoscopic RFA has been demonstrated. Studies discussed in the narrative review also showed that the mean time to discharge was 10.7 hours, with a mean of 9.0 days to return to normal activity and 6.5 days to return to work. For this patient, this is very beneficial because one of her top concerns is being able to return to work as soon as possible. The main side effects of laparoscopic RFA include fever, pain, and leukocytosis. A recent meta-analysis study published in the Journal of Minimally Invasive Gynecology demonstrated that the total adverse event rate of laparoscopic RFA was only 1.78%, including more serious events such as postoperative necrosis and peritonitis [6]. In case this patient desires to have more children, this procedure would be a safe option for her. In a case series study published in the Journal of Minimally Invasive Gynecology, 86.7% of pregnant women who had undergone laparoscopic RFA had full-term live births [6].

The second treatment option was open abdominal myomectomy [7]. Although this patient’s fibroid is a good candidate for the procedure based on location and size, it’s not the most convenient for her based on the increased risk of adverse events and prolonged recovery time [7]. This procedure involves open abdominal surgery to remove uterine fibroids [7]. Although this option could eliminate the fibroid as opposed to being reduced in size as with Acessa, the patient chose to do laparoscopic RFA due to increased advantages vs disadvantages in her case particularly [8]. A retrospective study in PubMed has shown that open myomectomies have considerably more blood loss, require a longer hospital stay, and have a longer recovery time when compared to a laparoscopic technique such as Acessa [8]. Infections, postoperative pain, and pelvic adhesions are also more common with open abdominal myomectomy when compared to a laparoscopic method [8]. In terms of pregnancy-associated risks, there is no significant difference in comparison to laparoscopic ablation, except that open myomectomy is associated with higher rates of cesarean deliveries and possible uterine rupture [8].

## Conclusions

In conclusion, this case report underscores the complexity of diagnosing and managing AUB. The patient's history, examination findings, and imaging results led to the identification of a uterine leiomyoma. The chosen treatment, laparoscopic radiofrequency ablation (Acessa), was selected for its favorable risk-benefit profile and alignment with the patient's preferences. This case highlights the importance of a multidisciplinary approach in providing personalized and effective care for women with AUB.
